# In What Does the Improvement in Dentistry during the Last Fifteen Years Consist?

**Published:** 1876-09

**Authors:** G. V. Black

**Affiliations:** Jacksonville.


### ARTICLE IV.

### *In what does the Improvement in Dentistry during the last Fifteen Years Consist?*

BY G. V. BLACK, JACKSONVILLE.

It is perhaps as well to occasionally cast a look over the past, and scan closely the principal landmarks by which we have passed in gaining the present, and determine from them, if we can, the best course for the future. We all doubtless believe that we have been making rapid improvement, during the last fifteen years, in all departments of our specialty; that the barriers which stood in our way have been continually breaking down before us; and that although the path has sometimes been rough and toilsome, our course has ever been onward and upward. Has this been true, or have we been like the mariner in the dark night when the spray, leaping from his prow, leads him to believe he is making good speed, but who, when morning dawns, finds that he is stemming a contrary current which overcomes all progress. No intelligent dentist of long experience can look back over his field of labor and fail to appreciate that a very great advance has been made. Yet, on looking over our literature published fifteen years ago, we find that much of the practice of to-day is the same as it was then. We filled teeth with gold, tin and amalgam, and the discussion over the use of the heated amalgam waxed warm then, as now. The merits of cohesive and soft foils were sharply discussed, and the forms of the rope, the pellet and the cylinder each had their advocates. Pulps were devitalized and pulps were capped, though this latter operation was not an admitted success in any particular class of cases. The filling of roots was well done and well explained, both theoretically and practically, though the modes had not found a place in the text-books. So we might go on through most of the operations which we perform to-day, and find that they were performed then much as now, with the same end in view—under the same circumstances—in keeping with the same theories, the differences being mainly in the manner of doing the work.

In what then, have we progressed? While it is true that teeth were filled then much as now, and with the same materials, it is also true that all these materials have undergone improvements in every stage and progress of their reparation. Every instrument has undergone a modificaon, a change for the better, or been supplanted by instruments made on new plans altogether; while every particular portion of the operation of making a filling has undergone modifications and improvement—so much so, indeed, that the dentist of fifteen years ago would not be able to comprehend the manipulations nor the instruments of the dentist of to-day.

This, then, is mechanical improvement; and the fact must stand out prominently to every thinking man who carefully reviews the last fifteen years of dentistry, that the great advance in practice of which we boast is principally in the mechanical department of our specialty. (When we speak of mechanics in this paper, we make no reference to the construction of artificial teeth.) The mechanics of dentistry, then, have been advanced along the whole line—not only have the old instruments been improved and supplanted by those made on different plans, but instruments and appliances in themselves entirely new to the profession, involving different modes of procedure, have been added. One of the earliest of these was the hand-mallet, and its convenient little series of automatic mallets, without which we doubt if the hand mallet would easily have attained the place it now occupies. Very many of the profession first learned the value of mallet pressure through the use of the automatic mallet, and thus gained the courage to employ assistants to use the hand-mallet, which placed at the chair another helper of great worth, outside of the use of the mallet, which was certainly the cause of their general introduction among us. The next and by far the most important improvement within the time of which we write, is the rubber dam, introduced by Dr. Barnum. This improvement upon the old modes far outstrips all others, and has done most toward the mechanical perfection of our operations, while it has rendered practicable a large advance in their range. It has brought forth the utilization of large numbers of these theoretical possibilities but practical impossibilities of former times. This, even did not find its way into general use without much trouble and no little opposition. And now many of us can look back over our fruitless endeavors to adjust it, and wonder how we could have been so awkward in its management. Now, however, having become skilled in its use, we only remember our former troubles as good jokes which serve to provoke mirth, and cause no regrets. This should teach us never in the future to give up a thing that promises good results because difficulties arise from our inexperience in the use of it. The next advance steps, that stand out sharp and clear are the introduction of the burring machine, or dental lathe, which is another example of the complete displacement of an old method by a new, and is purely a mechanical advance. Indeed, the instrument is simply one of the conveniences of the operating room, but it is one whose convenience is so very great that it becomes an actual necessity. Almost every operation that can be performed with it can also be performed without it, but every operation is performed better by its use, and a load of labor and suffering is lifted from both operator and patient.

There are many other distinct mechanical improvements that might be noticed as elements of our substantial advancement in the last fifteen years, but we have not the time to present more in this paper. Our advancement in this department, while it may not be all that could be desired, is certainly more than the most sanguine could have expected, and it seems to us now much as though it would be impossible to make an equal advance in the fifteen years to come.

We must now endeavor to speak of our advancement in pathology and therapeutics, and we admit, in the beginning, the great difficulty of properly treating this, the most important subject with which we have to deal. The subject is not only different in itself, but it is rendered doubly so by the desultory manner in which it is taught in both our journal literature and text-books. It would certainly seem that the improvement in our knowledge of pathological conditions and their merits should be the great end for which our profession should strive ; yet, singularly enough this department seems not to have received that earnest and co-operative thought which has been given to the mechanical department. While the whole profession, as it were, has been a unit in the strife for mechanical improvement—on the watch for the last new instrument, and ever ready with their endeavors at their creation, but few have given their energies to the more purely scientific branches, and even among these there has been less harmony in determination and thought, less earnestness and stability of purpose. Nevertheless, we have not stood still, although the advance made is not such as can be definitely taken hold of and printed out as that in the mechanical department, yet there is now among our best men more certainty in diagnosis and the adaptation of ends to meet the needs of pathological conditions ; and we have had in this field a few faithful workers, whose labors have not been without reward. There is one point in this field, and we believe but one, the treatment of exposed pulps. which stands out sharp and clear, as making a stride forward in the adaptation of means to meet the needs of a diseased condition, but even this seems to have been largely dependent on the introduction of a new material, the oxy-chloride of zinc, which we believe first made its appearance in 1858, but was not established as a material for capping exposed pulps until ten years later. It was used for this purpose, however, by the writer as early as 1859, and perhaps by others to a limited extent in the years following. But it was first brought to the general notice of the profession by Wm. H. Atkinson, of New York through our societies some years afterward, and was quickly adopted by many members of the profession, while it was very sharply opposed by others. Much discussion as to the conditions and needs of exposed pulps was engaged in throughout the land, which has resulted in a general understanding of the subject, which could not have arisen from any other cause, or in any other manner. The information resulting from this general and persistent inquiry by the profession *en-masse* constitutes the principal vantage ground we have gained, rather than the material introduced; for if oxy-chloride of zinc was banished from existence to-day, our knowledge of the conditions of success in treating exposed pulps gained through this discussion would enable us to adapt means to the end without much loss of time. On reviewing the introduction of this material we cannot but admit that it was badly abused by its friends as well as its enemies, and that it was the cause of much suffering and actual injury to patients, and the source of many regrets to the operators before its proper use was learned, and the classes and conditions to which it was applicable determined. Now, however, it may be said to have measurably emerged from its period of misuse, and marks one of the great improvements in modern dentistry. Aside from this, we can not now recall much that can be insolated and treated as distinct improvement. There is nevertheless a much more definite understanding of many of the diseased conditions, arising rather, as it were, from the intimacy of long experience than from the appreciation of new truths. Our adaptation of fillings to particular cases for the protection of teeth from the ravages of caries may be better, in a pathological sense, but I know of no new fact that has arisen. Pericementitis is treated upon the same plan now as then and alveolar abscess is cured by the same means. Absorption of the gums and alveolar processes baffle our best endeavors for its arrest, now as then.

In giving expression to these thoughts, we are necessarily confined to the consideration of the information attained then and now by those best versed in the treatment of the diseases with which we meet, but when we come to consider the professional status of dentists, *en-masse*, the whole matter is changed, and the difference then and now is very prominent, and shows a great stride forward, which is the result of the dissemination of knowlege among the members of our profession. At the time of which we speak, the number of well-informed men was comparatively small, and confined for the most part to our larger cities. It was the period of the birth of our dental societies, the largest portion of which came into existence during the decade preceding the year 1866. This seems to have been a period of general awakening, of general organization, for united effort. The few societies organized previous to that period and necessarily covering a large territory in order to obtain a respectable membership, had begun to make their influence felt throughout the land. New societies sprung into existence all over the country, in every state, in every city, everywhere, where any considerable number could be brought together. Now our country is thickly populated with earnest, working societies, and almost every section is reached by them, and every dentist is made to feel their influence, whether he be a member or not. With these has been combined the influence of our dental journals, which have increased in number and worth, and which have largely increased their circulation and influence. I think I am safe in saying that where one dental journal was taken and read fifteen years ago, fifty are taken and read to-day ; and thus they have become powerful instruments for the dissemination of useful knowledge among us. These have also been powerfully supplemented again by our dental schools, which have turned out hundreds of young men fitted for practice, and have done a good work in the dissemination of useful knowledge. Their influence has served to impress upon the minds of young men about entering the profession the necessity of a thorough preparatory course of study, even among those who have not availed themselves of a collegiate course; while they awakened those already in practice to greater efforts to become thoroughly competent. All these influences have united happily in one grand impulse for the dissemination of useful knowledge. The facts known to our best men have been spread and pushed out among the less informed, until the members of those well-fitted for the duties of the profession are vastly increased, until every state has its scores of teachers earnestly laboring through our societies, through our journals, and by their personal contact with their brethren, for the spread and utilization of useful information and the general elevation of the status of our superiority. The bars of ignorance, solecism and secrecy which were once so rife amongst us have been continually crumbling away before these combined powers, and dentists everywhere are being awakened to a higher appreciation of the duties they owe to themselves, the public, and their profession. Thus it is that we have the appearance of a greater improvement in the adaptation of means to meet the needs of diseased conditions than actually exists, when we consider the subject from facts known to our advanced men fifteen years ago. While we have not developed many new facts or theories in the department of pathology and therapeutics, we have shown a most excellent work in the spread and utilization of the facts and theories then known. Where we had but few who were able to judge nicely of the best adaptations of fillings for the protection of the teeth in different cases, or skilled in obtaining the best conditions for the performance of operations, we now have many. From this view of the subject, the advance in our knowledge, as a class, is very great. As a profession, however, after admitting all this, it still seems to be true that our manipulative ability has distanced our ability in adaptation ; or in other words, we too often perform splendid operations that are ill-timed, and mis-adapted to the conditions present. To correct this we need a more thorough knowledge, and a higher appreciation of general pathology and therapeutics as a basis for the study and treatment of the diseases met with in our specialty, that we may attain to broader views, and the more nicely adapt our manipulative powers to meet the needs of our patients. We need more united and continuous effort in this direction —*Illinois State Dental Society Transactions.*

